# Supplementary material for: Transcriptomic Insights into Mechanisms of Early Seed Maturation in the Garden Pea (Pisum sativum L.)
Source: Cells. 2020 Mar 23;9(3):779. doi: 10.3390/cells9030779 (PMC7140803; doi:10.3390/cells9030779)

Zhewan−1(25) vs Zhon

Global Summary

%DE = 0.83

genes with fdr < 0.2 = 512 (344 + / 168 −)

genes with fdr < 0.1 = 423 (267 + / 156 −)

genes with fdr < 0.05 = 295 (180 + / 115 −)

genes with fdr < 0.01 = 229 (134 + / 95 −)

genes in genesets = 0

<FC> = 0

<t−score> = −73192.53

<p−value> = 0

<fdr> = 0.17

Global Genelist

Rank	ID	log(FC)	p−value	fdr	Metagene	Description
1	TRINITY_DN1	−15.24	0e+00	0.0000000[illegible]	[illegible]0 x 1	
2	TRINITY_DN7	−0.44	0e+00	0.0000000[illegible]	[illegible]0 x 20	
3	TRINITY_DN3	0.44	0e+00	0.0000000[illegible]	[illegible]0 x 1	
4	TRINITY_DN2	−5.84	5e−09	0.0000000[illegible]	[illegible]0 x 1	
5	comp40744_c	−0.07	1e−08	0.000000[illegible]	[illegible]3 x 13	
6	TRINITY_DN5	−0.25	2e−08	0.000002[illegible]	[illegible]2 x 3	
7	TRINITY_DN1	5.21	4e−08	0.000002[illegible]	[illegible] x 1	
8	TRINITY_DN3	−0.14	6e−08	0.000002[illegible]	[illegible]4 x 8	
9	TRINITY_DN1	0	7e−08	0.000002[illegible]	[illegible] x 11	
10	TRINITY_DN5	0.45	9e−08	0.000008[illegible]	[illegible] x 20	
11	TRINITY_DN3	17.71	3e−07	0.000008[illegible]	[illegible] x 20	
12	TRINITY_DN1	−0.01	3e−07	0.000008[illegible]	[illegible] x 12	
13	TRINITY_DN1	−0.29	4e−07	0.0000008[illegible]	[illegible]8 x 10	
14	TRINITY_DN6	−1.76	4e−07	0.0000008[illegible]	[illegible]0 x 1	
15	TRINITY_DN4	−0.18	5e−07	0.000020[illegible]	[illegible] x 2	
16	TRINITY_DN2	0.06	7e−07	0.000020[illegible]	[illegible] x 13	
17	TRINITY_DN5	0.08	9e−07	0.000024[illegible]	[illegible] x 3	
18	TRINITY_DN3	−0.32	1e−06	0.000024[illegible]	[illegible]9 x 10	
19	TRINITY_DN1	0.02	2e−06	0.000024[illegible]	[illegible] x 9	
20	TRINITY_DN4	−1.51	2e−06	0.000024[illegible]	[illegible]8 x 20	

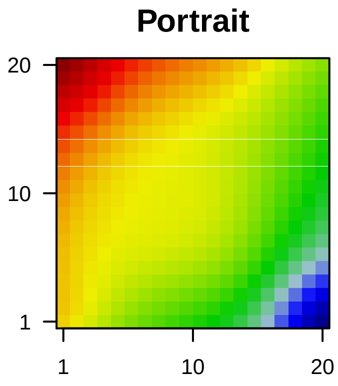

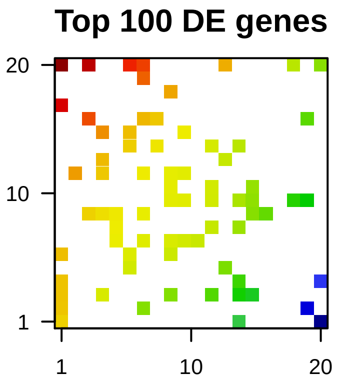

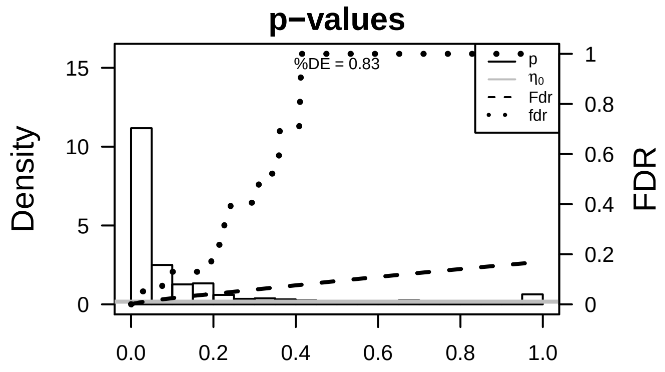

Supplement: Supplementary file 1 [file cells-09-00779-s001.zip › Supplementary materials/FileS3.zip!/FileS2/Hormones - Results/Summary Sheets - Differences/Reports/Zhewan.1.25..vs.Zhongwan.6.25..pdf]
